# Moxibustion for ulcerative colitis: a systematic review and meta-analysis

**DOI:** 10.1186/1471-230X-10-36

**Published:** 2010-04-07

**Authors:** Dong-Hyo Lee, Jong-In Kim, Myeong Soo Lee, Tae-Young Choi, Sun-Mi Choi, Edzard Ernst

**Affiliations:** 1 Division of Standard Research, Korea Institute of Oriental Medicine, Daejeon, Korea; 2 Clinical Research Center, Wonkwang University Sanbon Oriental Medical Center, Gunpo, Korea; 3 Department of Acupuncture and Moxibustion, College of Oriental Medicine, Kyung Hee University Medical Center, Seoul, Korea; 4 Complementary Medicine, Peninsula Medical School, Universities of Exeter & Plymouth, Exeter, UK

## Abstract

**Background:**

Complementary and alternative medicine (CAM) is increasingly used for treatment of inflammatory bowel disease (IBD). Acupuncture-type treatments are among the most popular options. Several studies have reported that moxibustion is effective in ulcerative colitis (UC). The objective of this review was to assess the clinical evidence for or against moxibustion as a treatment for UC.

**Methods:**

We searched the literature using 18 databases from their inception to February 10, 2010, without language restrictions. We included randomized clinical trials (RCTs), in which human patients with UC were treated with moxibustion. Studies were included if they were placebo-controlled or controlled against a drug therapy or no treatment group. The methodological quality of all RCTs was assessed using the Cochrane risk of bias.

**Results:**

In total, five RCTs were included. All were of low methodological quality. They compared the effects of moxibustion with conventional drug therapy. Three tested moxibustion against sulfasalazine and two against sulfasalazine plus other drugs. A meta-analysis of five RCTs showed favorable effects of moxibustion on the response rate compared to conventional drug therapy (n = 407; risk ratio = 1.24, 95% CI = 1.11 to 1.38; P < 0.0001; heterogeneity: I^2 ^= 16%).

**Conclusions:**

Current evidence is insufficient to show that moxibustion is an effective treatment of UC. Most of included trials had high risk of bias. More rigorous studies seem warranted.

## Background

Ulcerative colitis (UC) is a common, chronic idiopathic inflammatory bowel disease (IBD) [1]. Patients typically present with bloody diarrhea, passage of pus, mucus, or both, and abdominal cramping during bowel movements [2]. UC often requires lifelong medication, but poor adherence to medication has been an important barrier to successful management. Relapse rates are high, and the risk of colorectal cancer has increased [3,4].

Complementary and alternative medicine (CAM) is increasingly used for treatment of IBD [5], and acupuncture and moxibustion are particularly popular options. Moxibustion is a traditional Oriental medicine that uses the heat generated by burning herbal preparations containing *Artemisia vulgaris *to stimulate acupuncture points. Direct moxibustion is applied directly to the skin surface at an area around an acupuncture point, whereas indirect moxibustion is performed with some insulating materials (e.g., ginger, salts) placed between the moxa cone and the skin [6]. The heat is then used to warm the skin at the acupuncture point. Several observational studies have reported that moxibustion is effective in UC [7-9], and animal studies have suggested beneficial effects [10,11].

A recent systematic review included clinical trials of acupuncture for gastrointestinal disorders, showing positive effects of acupuncture [12]. Considering that moxibustion is closely related to acupuncture, it seems pertinent to evaluate the effectiveness of this therapy under these conditions. Currently, no systematic review of moxibustion for UC is available. Hence, it was the aim of this systematic review to summarize and critically evaluate the evidence for or against the effectiveness of moxibustion as a symptomatic treatment for UC.

## Methods

### Data sources

The following databases were searched from their inception through February 10, 2010 (first searched in July 2009): MEDLINE, AMED, EMBASE, CINHAL, PsycInfo, five Korean Medical Databases (Korean Studies Information, DBPIA, Korea Institute of Science and Technology Information, KoreaMed, and Research Information Centre for Health Database), four Chinese Medical Databases (China Academic Journal, Century Journal Project, China Doctor/Master Dissertation Full Text Database, and China Proceedings Conference Full Text Database), The Cochrane Library 2010, Issue 1, and three Japanese electronic databases. The search terms used were "moxibustion" or "ulcerative" in Korean, Chinese, or English. Reference lists of all obtained papers were searched. We also performed electronic searches of relevant journals (FACT [Focus on Alternative and Complementary Therapies] and Research in Complementary Medicine [Forschende Komplementarmedizin] up to Februrary 2010). Additionally, reference lists of all obtained papers were searched, and our own personal files were manually searched as well. Hardcopies of all potentially relevant articles were obtained and read in full. In addition, the proceedings of United European Gastroenterology Week (UEGW) from 2006 to 2009 and Digestive Disease Week (DDW) from 2008 and 2009 were searched for other relevant articles.

### Study selection

We included RCTs in which human patients with UC were treated with moxibustion. The studies were included if they were placebo-controlled or controlled against a conventional treatment, including drug therapy and another active treatment, or against no treatment. Trials testing the effectiveness of moxibustion combined with other therapies were excluded. Dissertations and abstracts were included when they contained sufficient details.

### Data extraction, quality, and validity assessment

All articles were read by two independent reviewers (DHL, JIK), who extracted data from the articles according to predefined criteria (Table 1). Risk of bias was assessed using the Cochrane classification in four criteria: sequence generation, incomplete outcome measures, blinding, and allocation concealment [13]. Considering that it is virtually impossible to blind therapists to the use of moxibustion, we assessed patient and assessor blinding separately. Disagreements were resolved by discussion between the two reviewers (DHL, JIK), with the opinion of a third reviewer (MSL) being sought if necessary. There was no disagreement between the two reviewers about the risk of bias.

**Table 1:** Summary of randomized clinical studies of moxibustion for ulcerative colitis with parallel design

First author (Year) [ref]	Sample size (M/F) Duration of disease Age (range) Setting* (author's affiliation)	Experimental intervention	Control intervention	Response rate^§^ (basis of assessment)
Wen (2003) [16]	69 (35/34) (A) 6 mon-14 yrs (B) 4 mon-16 yrs 23-69 yrs n.r. (TCM hospital)	(A) Moxa [once daily for 12 days (1 session), 3 day intervals between courses, total 6 sessions n = 39] Indirect	(B) Sulfasalazine (SASP, oral, 1 g × 4/d, for 3 months, n = 30)	A(89.8%, 35/39); B(66.8%, 20/30) P < 0.05 (Physician's assessment)
Wu (1999) [17]	151 (n.r.) (A) 6 mon-18 yrs (B) 4 mon-16 yrs (C) 4 mon-17 yrs 25-70 yrs n.r. (TCM institute and Western hospital)	(A) Moxa I [once daily for 12 days (1 session), 3 day intervals between courses, total 6 sessions n = 65] (B) Moxa II(same as A, n = 56) Indirect	(C) Sulfasalazine (SASP only, oral, early: 1 g × 4/d, firmly: 0.5 g × 4/d, for 3 months, n = 30)	A(92.3%, 60/65); B(89.3%, 50/56); C (66.7%, 20/30) A, C: P < 0.01; B, C: P < 0.05 (Physician's assessment)
Ding (2009) [18]	61 (32/29) (A) 3 mon-20 yrs (B) 3 mon-17 yrs 19-71 yrs n.r. (Western hospital)	(A) Moxa [20 min, once daily for 2 months, n = 30] Indirect (ginger)	(B) Sulphasalazine (oral, 1 g × 4/d, for 1 month, n = 31)	A(100%, 30/30); B(90.3%, 28/31) P < 0.05 (Physician's assessment, endoscopy)
Wang (2006) [19]	60 (28/32) (A) 0.5-12 yrs (B) 0.6-13 yrs 27-54 yrs TCM hospital and private clinics (TCM hospital and private clinics)	(A) Moxa [once daily for 12 days (1 session), 3 day intervals between courses, total 3 sessions n = 30] Indirect	(B) Sulphasalazine (1.0 g × 4/d) and Metronidazole (0.2 g × 3/d), oral, [once daily for 10 days (1 session), 3 day intervals between courses, total 3 sessions n = 30]	A(86.7%, 26/30); B(66.7%, 20/30) P < 0.05 (Physician's assessment, endoscopy)
Zhou (2003) [20]	66 (31/35) 2-8 yrs 19-50 yrs n.r. (TCM hospital)	(A) Moxa [once daily for 10 days (1 session), 3 day intervals between courses, total 3 sessions n = 34] Indirect (ginger)	(B) Sulfasalazine (SASP, oral, 0.5 g × 4/d), for 30 days, n = 32] plus Prednisone [(oral, 10 mg × 4/d but reduce to 10 mg/d if getting a more stable state)	A(97.1%, 33/34); B(71.9%, 23/32) P < 0.05 (Physician's assessment, endoscopy)

Sulfasalazine: Anti-inflammatory, Metronidazole: Antibiotic, Prednisone: A synthetic anti-inflammatory glucocorticoid derived from cortisone,

SASP: salicylazosulfapyridine, ACTH: Adreno-Cortico Tropin Hormone, ACH: Adreno-Cortical Hormone

*The most of trials didn't describe the place of remision or neing treaed for active disease in the text. Alternatevely, we report the affiliation of the authors in the brackets.

^§^Trial divided into three or four categories, including (1) recovery, (2) marked improvement, (3) improvement, and (4) no change in terms of symptom and results of endoscopy.

### Outcome measures and data synthesis

All clinical endpoints were considered, but the main outcome measure was the response rate for treating symptoms in patients with UC. We did not evaluate the outcomes related to immunological or other surrogate endpoints. The differences between the intervention and control groups were assessed. Relative risk (RR) and 95% confidence intervals (CIs) were calculated using Cochrane Collaboration's software (Review Manager [RevMan] Version 5.0 for Windows. Copenhagen: The Nordic Cochrane Center). Chi-square and Higgins I^2 ^tests were used to assess heterogeneity. Where more than 10 studies were available, we assessed publication bias using a funnel plot or Egger's regression test [14,15].

## Results

### Study description

**Figure 1:**
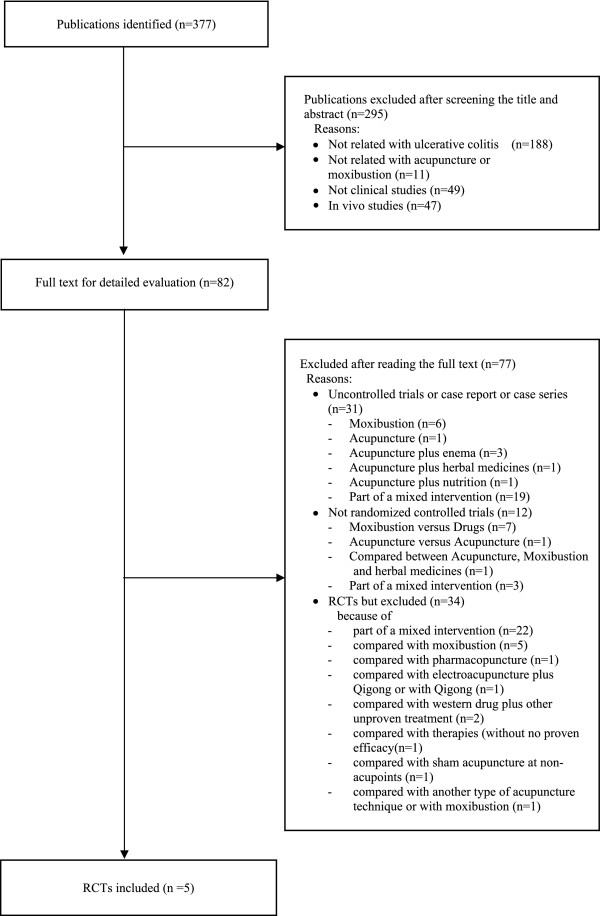
**Flow chart of trial selection process**. RCT: randomized clinical trial.

Our searches identified 377 potentially relevant studies, of which 5 met our inclusion criteria (Figure 1). The key data from all included RCTs are listed in Table 1[16-20]. All of the RCTs originated from China. Four adopted a two-arm parallel group design [16,18-20], and one adopted a three-arm parallel group design [17]. In all RCTs, the treatment was based on the principles of traditional Chinese medicine (TCM) as the rationale for selecting the acupuncture point. Selected acupuncture points from all trials and other information related to treatments are listed in sufficient detail in Table 2. Most of the included studies used response rate for each intervention, and outcomes were typically divided into four categories, including (1) recovery, (2) marked improvement, (3) improvement, and (4) no change. These were based on the both physician's assessment and the results of endoscopy in three trials [18-20], while the other two studies employed physician assessment [16,17]. The setting was described in one trial [19], while the others did not report such details [16-18,20].

**Table 2:** Summary of treatment points and other information related to treatment

First author (Year) [ref], Country	Treatment points	Rationales	Adverse events
Wen (2003)[16] China	Fixed points: (10 points: A and B were alternately treated) A) CV12, CV6, ST36 B) BL25, ST25, ST37 Possible additional points(individualized): Spleen and stomach deficiency-BL20; Damp heat accumulation-CV9; Liver stagnation and spleen deficiency-BL18, BL20; Spleen and kidney yang deficiency-BL23, CV4	TCM theory and modern scientific evidence	n.r.
Wu (1999)[17] China	Fixed points: (10 points: A and B were alternately treated) A) CV12, CV6, ST36 B) BL25, ST25, ST37 Possible additional points(individualized): Spleen and stomach deficiency-BL20; Damp heat accumulation-CV9; Liver stagnation and spleen deficiency-BL18, BL20; Spleen and kidney yang deficiency-CV4; Constipation-KI15; Pyemia(severe)-SP1 Medicinal cake consist of: Moxibustion Group I-Aconiti Lateralis Radix Preparata, Cinnamomi Cortex Spissus, Salviae Miltiorrhizae Radix, Carthami Flos, Aucklandiae Radix, Coptidis Rhizoma, etc. Moxibustion Group II-Santali Albi Lignum, Syzygii Flos, Bomeolum, Zanthoxyli Fructus, etc.	TCM theory and clinical experiences	n.r.
Ding (2009) [18] China	Fixed points: (8 points) BL13, BL20, BL23, BL25	TCM theory	n.r.
Wang (2006)[19] China	Fixed points: (1 point) CV8	TCM theory, previous studies and anatomical features	n.r.
Zhou (2003)[20] China	Fixed points: (12 points: A and B were alternately treated) A) CV12, ST25, ST36, BL20, BL26 B) CV12, ST25, ST36, BL23, GV4 A), B): alternately every day	TCM theory	None

TCM: Traditional Chinese Medicine; n.r.: not reported

### Risk of bias

All of the included RCTs had high risk of bias. Of the five included RCTs, four did not describe the sequence generation. One RCT employed the methods of sequence generation incorrectly [19]. None of the studies described any attempt to blind assessors. All of the RCTs reported incomplete outcome measures and allocation concealment. Adverse events were mentioned only in one RCT [20].

### Description of individual studies

Wen [16] conducted an RCT assessing the effectiveness of moxibustion on symptoms of UC patients. Sixty-nine patients were divided randomly into two parallel groups: moxibustion (n = 39) and sulfasalazine (n = 30). At the end of the treatment period, 89.8% of patients from the experimental group had improved, while the corresponding figure in the control group was 66.8% (P < 0.05).

Wu and co-workers [17] tested the effects of moxibustion in 151 patients who were divided randomly into a three parallel groups: moxibustion group I (details of composition are listed in Table 2, n = 65), moxibustion group II (details of composition are listed in Table 2, n = 56), and a sulfasalazine group (n = 30). The response rate was 92.3% in moxibustion group I, 89.3% in moxibustion group II, and 66.7% in the sulfasalazine group.

Ding and co-workers [18] conducted an RCT to test the therapeutic effect of ginger moxibustion on Yang deficiency of the spleen and kidney in patients with UC. Sixty-one patients were randomly divided into two groups. Thirty patients were given ginger moxibustion, while in the control group 31 patients were administered sulfasalazine. The effectiveness rate was 100%, and the curative effect was 90.0% in the experimental group. These rates were significantly better than in the control group (90.3% and 35.5%, respectively; *P *< 0.01).

Wang and co-workers [19] conducted an RCT to test the therapeutic effect of moxibustion at Shenque (CV8) on UC. Sixty patients were randomly divided into two parallel groups: moxibustion (n = 30) and sulfasalazine plus metronidazole (n = 30). The total response rate was 86.7% in the moxibustion group and 66.7% in the control group.

Zhou [20] randomized 60 patients into two parallel groups: moxibustion (n = 34) and prednisone plus sulfasalazine (n = 32). Response rates were measured by symptoms, fiber colonoscopy, endoscopy, and pathological examination. The total effective rate was 97.1% in the moxibustion group and 71.9% in the control group (P < 0.05). No adverse event was reported.

### Meta-analysis

**Figure 2:**
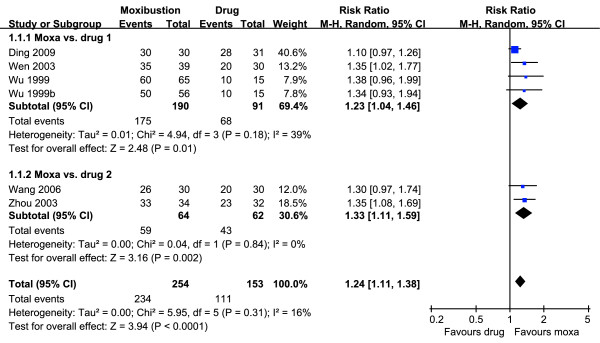
**Forest plot of moxibustion for ulcerative colitis compared to conventional drug**. Moxa: moxibustion.

The meta-analysis of the five RCTs [16-20] suggested favorable effects of moxibustion on the RR compared with conventional drug therapy (n = 407; RR = 1.24, 95% CI = 1.11 to 1.38; P < 0.0001; heterogeneity: I^2 ^= 16%, Figure 2). A subgroup analysis [16-18] also demonstrated beneficial effects of moxibustion compared to sulfasalazine alone (n = 281; RR = 1.23, 95% CI = 1.04 to 1.46; P = 0.01; heterogeneity: I^2 ^= 39%). A further analysis [19,20] showed favorable effects of moxibustion compared to sulfasalazine plus metronidazole or prednisone (n = 126; RR = 1.33, 95% CI = 1.11 to 1.59; P = 0.002; heterogeneity: I^2 ^= 0%).

We could not assess publication bias because of the low number of studies [14,15].

## Discussion

Few RCTs have tested the effects of moxibustion for UC, and none of the existing trials were methodologically rigorous. Our meta-analysis suggests that moxibustion is effective; however, the number of trials, their quality and the total sample size are too low to allow firm conclusions.

Cochrane criteria were used to quantify the likelihood of bias inherent in the studies based on the description of sequence generation, incomplete outcome measures, blinding and allocation concealment. All included RCTs had high risk of bias. Low-quality trials (high risk of biased trials) are more likely to overestimate the effect size [21]. None of the RCTs described attempts to blind patient or assessors, dropouts and withdrawals, or allocation concealment. In addition, all RCTs failed to report details regarding ethical approval. Thus, the reliability of the evidence presented here is clearly limited.

It has been repeatedly noted that trials originating from China are rarely, if ever, negative [22]. All of the included RCTs originated from China. The absence of negative results is a largely unexplained phenomenon. Whatever the causes, it does not increase our confidence in these studies.

In the absence of reliable data from controlled clinical trials, other types of evidence might be helpful. The results of all of the uncontrolled trials or case reports implied that acupuncture improves symptoms of UC. Unfortunately, such data are highly susceptible to bias; hence, they provide little useful information on the specific effects of moxibustion as a therapeutic intervention for UC.

By considering moxibustion as a type of therapeutic intervention by stimulating acupuncture points, we could include three other RCTs (Additional file 1). Three RCTs tested acupuncture plus moxibustion versus conventional medicines [23-25]. Two RCTs compared acupuncture plus moxibustion with sulfasalazine [23,24]. One RCT reported favorable effects of acupuncture plus moxibustion compared to drug therapy [23], while the two other RCTs failed to do so [24,25]. The pooling of these three RCTs failed to showed favorable effects of moxibustion plus acupuncture for UC compared to drug therapy (n = 273; RR = 1.15, 95% CIs = 0.91 to 1.46; P = 0.24), although marked heterogeneity was observed in this model (χ^2 ^= 15.27; P = 0.0005; I^2 ^= 87%; Additional file 2).

Assuming that moxibustion is a beneficial treatment for UC, its mechanisms may be of interest. These may include improvement of immune function or effects on intestinal mucosal morphology and expression of GH and IGF-I [10,26]. None of these theories, however, are currently more than speculation.

Limitations of our systematic review (and indeed systematic reviews in general) pertain to the potential incompleteness of the evidence reviewed. We aimed to identify all studies on the subject. The distorting effects of publication bias and location bias on systematic reviews and meta-analysis are well documented [27-30]. In the present review, there were no restrictions on the review publication language, and a large number of different databases were searched. We are, therefore, confident that our search strategy located all relevant data on the subject. However, a degree of uncertainty remains. Further limitations include the paucity and the often suboptimal quality of the primary data. Additionally, all included RCTs that reported positive results came from China, one of the countries that produces virtually no negative results [31], a fact that casts some doubt on the validity of such data. All of the included studies were conducted in Asia, therefore making the conclusions limited to Asian populations. For other populations, independent replications are required. None of the RCTs included in our review were successful in minimizing bias. Collectively, these facts seriously limit the conclusiveness of our systematic review.

Future studies in UC treatment with moxibustion should emphasize adequate methods to permit RCTs and the use of pilot trials to help prepare appropriate RCTs. Long-term studies are also needed to determine the longevity of treatment effects. Moreover, a cost-analysis should be considered.

## Conclusion

The evidence that moxibustion is an effective treatment for UC is inconclusive. Even though the trial data are unanimously positive, too many important caveats exist to draw firm conclusions.

## Competing interests

The authors declare that they have no competing interests.

## Authors' contributions

DHL, JIK and MSL conceived the study design. DHL, JIK and MSL searched and selected the trials, extracted, analyzed and interpreted the data. DHL and MSL drafted the manuscript. TYC updated the search and the content of the review. SMC and EE helped with the study design and critically reviewed the manuscript. All authors read and approved the final version of the manuscript.

## Pre-publication history

The pre-publication history for this paper can be accessed here:

http://www.biomedcentral.com/1471-230X/10/36/prepub

## Supplementary Material

Additional file 1 **Summary of randomized clinical studies of moxibustion plus acupuncture for ulcerative colitis**. We smmarize 3 randiomized clinical trials of moxibustion plus acupuncture for ulcerative colitis compared with conventional drug therapies. Click here for file

Additional file 2 **Forest plot of moxibustion plus acupuncture for ulcerative colitis compared to conventional drug**. We pooled the response rate from 3 randiomized clinical trials of acupuncture plus moxibustion for ulcerative colitis compared with conventional drug therapies. Click here for file

## References

[B1] Duerr RH Taylor KD Brant SR Rioux JD Silverberg MS Daly MJ Steinhart AH Abraham C Regueiro M Griffiths A A genome-wide association study identifies IL23R as an inflammatory bowel disease gene Science 2006 314 1461 1463 10.1126/science.1135245 17068223 PMC4410764

[B2] Baumgart DC Sandborn WJ Inflammatory bowel disease: clinical aspects and established and evolving therapies Lancet 2007 369 1641 1657 10.1016/S0140-6736(07)60751-X 17499606

[B3] Kane SV Adherence issues in the treatment of ulcerative colitis Aliment Pharmacol Ther 2006 23 577 585 10.1111/j.1365-2036.2006.02809.x 16480396

[B4] Zhang Z Kennedy H Ulcerative colitis: current medical therapy and strategies for improving medication adherence Eur J Gastroenterol Hepatol 2009 21 1 8 10.1097/MEG.0b013e32830bfb88 19011573

[B5] Langmead L Rampton DS Complementary and alternative therapies for inflammatory bowel disease Aliment Pharmacol Ther 2006 23 341 349 10.1111/j.1365-2036.2006.02761.x 16422993

[B6] World Health Organization Western Pacific Region WHO International Standard Terminologies on Traditional Medicine in the Western Pacific Region 2007 Manila, Philippine: World Health Organization Western Pacific 251 254

[B7] Wu HG Zhou LB Shi DR Liu SM Liu HR Zhang BM Chen HP Zhang LS Morphological study on colonic pathology in ulcerative colitis treated by moxibustion World J Gastroenterol 2000 6 861 865 11819709 10.3748/wjg.v6.i6.861 PMC4728275

[B8] Zhou EH Liu HR Wu HG Shi Z Zhang W Zhu Y Shi DR Zhou S Down-regulation of protein and mRNA expression of IL-8 and ICAM-1 in colon tissue of ulcerative colitis patients by partition-herb moxibustion Dig Dis Sci 2008 54 2198 2206 10.1007/s10620-008-0620-4 19083096

[B9] Joos S Wildau N Kohnen R Szecsenyi J Schuppan D Willich SN Hahn EG Brinkhaus B Acupuncture and moxibustion in the treatment of ulcerative colitis: a randomized controlled study Scand J Gastroenterol 2006 41 1056 1063 10.1080/00365520600580688 16938719

[B10] Wu HG Liu HR Tan LY Gong YJ Shi Y Zhao TP Yi Y Yang Y Electroacupuncture and moxibustion promote neutrophil apoptosis and improve ulcerative colitis in rats Dig Dis Sci 2007 52 379 384 10.1007/s10620-006-9561-y 17211698

[B11] Liu Hr Tan Ly Wu Hg Zhu Y Zhao Cy Cui Yh Jiang B Wang Xm Effect of moxibustion on the synthesis and secretion of collagen by colonic fibroblasts in ulcerative colitis fibrosis rats J Acupuct Tuina Sci J Acupuct Tuina Sci 2008 6 4 7 10.1007/s11726-008-0004-5

[B12] Schneider A Streitberger K Joos S Acupuncture treatment in gastrointestinal diseases: a systematic review World J Gastroenterol 2007 13 3417 3424 17659687 10.3748/wjg.v13.i25.3417 PMC4146776

[B13] Higgins JPT Altman DG Higgins JPT, Green S Assessing risk of bias in included studies Cochrane Handbook for Systematic Reviews of Interventions 2008 West Sussex, England: Wiley-Blackwell 187 241 full_text

[B14] Borenstein M Hedges LV Higgins JPT Rothestein HR (Eds) Meta-regression 2009 West Sussexx, Uk: John Wiley & Sons, Ltd 187 203

[B15] Sterne JAC Egger M Moher D Higgins JPT, Green S Addressing reporting biases Cochrane Handbook for Systematic Reviews of Interventions 2008 West Sussex, England: Wiley-Blackwell 297 333 full_text

[B16] Wen L Clinical observation on the effect of moxibustion for chronic ulcerative colitis J Jiangxi College Trad Chin Med 2003 15 35 36

[B17] Wu H Tan W Chen H Shi Z Hua X Effect of moxibustion on ulcerative colitis and expression of HLA-DR antigen on epithelial cell of the colon Acupunct Research 1999 12 16

[B18] Ding H Wang H Zhang T Tao Y Effects of ginger moxibustion on 30 patients with asdthenic splenonephro-yang of ulcerative colitis Acta Academiae Medicine 2009 18 509 511

[B19] Wang S Li X Zhang L Xu Y Li Q Clinical study on drug-separated moxibustion at Shenque (CV 8) for treatment of ulcerative colitis Chin Acupunct Moxibustion 2006 26 97 99 16541855

[B20] Zhou J Clinical observation on the effect of moxibustion for ulcerative colitis in 34 cases Jiangsu J Trad Chin Med 2003 24 44 45

[B21] Moore A McQuay H Bandolier's Little Book of Making Sense of the Medical Evidence 2006 Oxford, UK: Oxford University Press

[B22] Tang JL Zhan SY Ernst E Review of randomised controlled trials of traditional Chinese medicine BMJ 1999 319 160 161 10406751 10.1136/bmj.319.7203.160 PMC28166

[B23] Ma X Acupuncture treatment for 76 cases of ulcerative colitis 265 J Trad Chin Med 2005 25 264 265 16447666

[B24] Yang C Yan H Observation of the efficacy of acupuncture and moxibustion in 62 cases of chronic colitis J Trad Chin Med 1999 19 111 114 10681867

[B25] Ma S Observation on the therapeutic effect of combined treatment of 60 cases of ulcerative colitis with acupuncture and moxibustion World J Acup-Mox 1999 9 24 26

[B26] Ma X Sang X Wu H Shi Z Liu H Wang X Effects of acupuncture and moxibustion on intestinal mucosa morphology and expressions of GH and IGF-I in ulcerative colitis rats Chin Arch Trad Chin Med 2007 25 1362 1365

[B27] Pittler MH Abbot NC Harkness EF Ernst E Location bias in controlled clinical trials of complementary/alternative therapies J Clin Epidemiol 2000 53 485 489 10.1016/S0895-4356(99)00220-6 10812320

[B28] Ernst E Pittler MH Alternative therapy bias Nature 1997 385 480 10.1038/385480c0 9020351

[B29] Egger M Smith GD Bias in location and selection of studies BMJ 1998 316 61 66 9451274 10.1136/bmj.316.7124.61 PMC2665334

[B30] Dickersin K The existence of publication bias and risk factors for its occurrence JAMA 1990 263 1385 1389 10.1001/jama.263.10.1385 2406472

[B31] Vickers A Goyal N Harland R Rees R Do certain countries produce only positive results? a systematic review of controlled trials Control Clin Trials 1998 19 159 166 10.1016/S0197-2456(97)00150-5 9551280

