# Supplementary material for: Moxibustion for ulcerative colitis: a systematic review and meta-analysis
Source: BMC Gastroenterol. 2010 Apr 7;10:36. doi: 10.1186/1471-230X-10-36 (PMC2864201; doi:10.1186/1471-230X-10-36)
Supplement: Additional file 1 — Summary of randomized clinical studies of moxibustion plus acupuncture for ulcerative colitis. We smmarize 3 randiomized clinical trials of moxibustion plus acupuncture for ulcerative colitis compared with conventional drug therapies. [file 1471-230X-10-36-S1.DOC]

**Additional file 1. Summary of randomized clinical studies of acupuncture plus moxibustion for ulcerative colitis**

| First author (Year) [ref] | Sample size (M/F)  Duration of disease  Age (range) | Experimental intervention | Control intervention | Response rate * | Treatment points | Rationales | Adverse events |
| --- | --- | --- | --- | --- | --- | --- | --- |
|  |  |  |  |  |  |  |  |
| Ma  (2005) [21]  China | 121 (67/54)  <10 yrs  21-69 yrs | (A) AT (30 min) plus Moxa [once daily for 20 days (1 session), 5 day intervals between courses, total 1-3 sessions n=76] | (B) Sulfasalazine [azulfidine, 1-2 g × 4/d, oral, once daily for 20 days (1 session), 5 day intervals between courses, total 1-3 sessions n=45] | A(96.1%, 73/76); B(71.1%, 32/45) | Fixed points: (11 points)  Acupuncture-ST37, ST25, CV4, ST36, CV12, BL25, GV1  Moxibustion-CV12, ST25, CV4 | TCM theory | n.r. |
| Yang  (1999) [22]  China | 62 (28/34)  (A) 6 mon-8 yrs  (B) 2 mon-10 yrs  35-50 yrs | (A) AT (30 min, once daily) plus Moxa [3 times daily for 10 days, 3 day intervals between courses n=32] | (B) Sulfasalazine [Salicylazosulfapyri-dinum, oral, 5 g/d for the stage of attack, q.i.d. and 2 g/d for the remission stage n=30] | A(31/32);  B(29/30)  P<0.05 | Fixed points: (5 points)  Acupuncture-ST25, CV4, ST36  Moxibustion-CV4, ST36, BL23, KI1 | TCM theory | Few side  effects |
| Ma  (1999) [23]  China | 90 (56/34)  (A) 6 mon-13 yrs  (B) 6 mon-10 yrs  23-68 yrs | (A) AT (30 min) plus Moxa [once daily for 30 days n=60] | (B) Sulfasalazine (Salicylazosulfapyri -dine, oral, 1 g × 4/d) and Metronidazole (IV, 0.5 g, once every day) for 30 days, n=30 | A(96.7%, 58/60); B(83.3%, 25/30)  P<0.05 | Fixed points: (16 points+Asihyeol: A and B  were alternately treated)  (A) Acupuncture-ST25, CV12, CV4, ST36,  LR3, Asihyeol (unfixed points)  Moxibustion-CV4  (B) Acupuncture-BL25, BL23, BL31, BL32  Moxibustion-BL31, BL32 | TCM theory | n.r. |

AT: acupuncture, Sulfasalazine: Anti-inflammatory, Metronidazole: Antibiotic

* Trial divided into three or four categories, including (1) recovery, (2) marked improvement, (3) improvement, and (4) no change in terms of symptom and results of endoscopy.
