# Supplementary material for: Moxibustion for ulcerative colitis: a systematic review and meta-analysis
Source: BMC Gastroenterol. 2010 Apr 7;10:36. doi: 10.1186/1471-230X-10-36 (PMC2864201; doi:10.1186/1471-230X-10-36)
Supplement: Additional file 2 — Forest plot of moxibustion plus acupuncture for ulcerative colitis compared to conventional drug. We pooled the response rate from 3 randiomized clinical trials of acupuncture plus moxibustion for ulcerative colitis compared with conventional drug therapies. [file 1471-230X-10-36-S2.DOC]

**Additional file 2.** Forest plot of moxibustion plus acupuncture for ulcerative colitis compared to conventional drug
